# Gene Therapy with Endogenous Inhibitors of Angiogenesis for Neovascular Age-Related Macular Degeneration: Beyond Anti-VEGF Therapy

**DOI:** 10.1155/2015/201726

**Published:** 2015-03-03

**Authors:** Selwyn M. Prea, Elsa C. Chan, Gregory J. Dusting, Algis J. Vingrys, Bang V. Bui, Guei-Sheung Liu

**Affiliations:** ^1^Department of Optometry & Vision Sciences, University of Melbourne, 4th Floor, Alice Hoy Building, 162 Monash Road, Parkville, VIC 3010, Australia; ^2^Centre for Eye Research Australia, Level 1, 32 Gisborne Street, East Melbourne, VIC 3002, Australia; ^3^Department of Ophthalmology, University of Melbourne, Level 1, 32 Gisborne Street, East Melbourne, VIC 3002, Australia

## Abstract

Age-related macular degeneration (AMD) is the leading cause of substantial and irreversible vision loss amongst elderly populations in industrialized countries. The advanced neovascular (or “wet”) form of the disease is responsible for severe and aggressive loss of central vision. Current treatments aim to seal off leaky blood vessels via laser therapy or to suppress vessel leakage and neovascular growth through intraocular injections of antibodies that target vascular endothelial growth factor (VEGF). However, the long-term success of anti-VEGF therapy can be hampered by limitations such as low or variable efficacy, high frequency of administration (usually monthly), potentially serious side effects, and, most importantly, loss of efficacy with prolonged treatment. Gene transfer of endogenous antiangiogenic proteins is an alternative approach that has the potential to provide long-term suppression of neovascularization and/or excessive vascular leakage in the eye. Preclinical studies of gene transfer in a large animal model have provided impressive preliminary results with a number of transgenes. In addition, a clinical trial in patients suffering from advanced neovascular AMD has provided proof-of-concept for successful gene transfer. In this mini review, we summarize current theories pertaining to the application of gene therapy for neovascular AMD and the potential benefits when used in conjunction with endogenous antiangiogenic proteins.

## 1. Introduction

Neovascular AMD is the most common cause of severe vision loss in patients over the age of 60 [1, 2]. End stage complications of dry and wet forms of AMD are geographic atrophy or choroidal neovascularization (CNV). Whilst both can lead to vision loss, the wet form is often the more deleterious of the two. CNV originates from the choriocapillaris, with new vessels penetrating through Bruch's membrane and growing into the subretinal pigment epithelium (RPE) and/or subretinal space. Newly formed vessels typically lack normal structural integrity, as evidenced by incomplete basement membrane and/or pericyte content, making them susceptible to leakage and hemorrhage [3]. Such leakage can cause retinal edema resulting in visual distortion and marked diminution of vision when the macula is involved. The recent availability of anti-VEGF monoclonal antibodies has revolutionized the treatment of neovascular AMD by preserving and even restoring vision in patients [4–6]. However, the systemic safety of repeated injections of anti-VEGF agents has raised concern, particularly with regards to reports of increased risk of hemorrhagic stroke [7, 8]. In addition, the loss of efficacy over time has brought into question the long-term benefits of anti-VEGF therapy [9].

The rapid advancement of gene therapy has placed this approach on the doorstep of clinical use in ophthalmology. Given that the eye is a particularly favourable organ for drug delivery, ocular use is likely to be among the most successful applications of this technique [10–13]. Positive results from a recent clinical trial and animal studies [14–18] suggest that gene transfer deserves more intensive study as a means to achieve local, sustained control of intraocular neovascularization (and possibly excessive vascular permeability) [19]. Indeed, gene-based approaches that can produce safe and long-term expression of one or more endogenous angiogenic inhibitors [20] would be a significant advance in the treatment of neovascular disease.

Gene transfer of endogenous angiogenic inhibitors such as pigment epithelium-derived factor (PEDF), endostatin, and angiostatin has provided beneficial effects in animal models [16] and in a Phase I clinical trial [21]. Other promising candidate transgene products for management of neovascular AMD include vasostatin [22], tissue inhibitor of metalloproteinases-3 (TIMP3) [17], plasminogen kringle 5 (K5) [23], and thrombospondin-1 [24]. This review seeks to briefly summarize current application of gene-based treatments for neovascular AMD and potential alternative treatments involving endogenous angiogenic inhibitors.

## 2. Pathogenesis and Current Treatment of Neovascular AMD

### 2.1. Pathogenesis of Neovascular AMD

The retina is metabolically unique in its specialisation for the capture of light and its transduction into an electrical signal. To support this activity there are extremely high energy needs, particularly for effective phototransduction and signal transmission as well as turnover of cellular membranes and phototransduction proteins. Not surprisingly, the retina is the most metabolically demanding of all the body's tissues [25]. The majority of the energy needed in the eye is required for neurotransmission and the maintenance of ionic gradients across the cell membrane. The remaining energy sustains vegetative function. In addition, much of the carbon substrates taken up as glucose into the eye are required for amino acid synthesis to support the turnover of photoreceptor outer segment membrane and membrane bound proteins. The retina also has a specialization, known as the macular, where a high density of cone photoreceptors allows for high spatial acuity. This specialization and the large metabolic burden make the retina particularly susceptible to metabolic insult and diseases that impact upon metabolic processes, such as AMD.

While many potential etiologies and pathological processes have been linked to AMD, our understanding of its development remains incomplete. In addition to aging as the major risk factor for AMD, other risk factors such as smoking, obesity, nutrition, and sunlight exposure have been strongly linked to AMD [26]. More recently, studies of the genetic basis of AMD have revealed variations in genes involved in lipid metabolism, inflammation, and oxidative stress can account for a substantial amount of AMD risk [26, 27].

Early AMD is characterised by the presence of extracellular debris beneath the retina known as drusen [28]. Early AMD can progress to advanced AMD, which has two types that include geographic atrophy AMD and neovascular AMD. Geographic atrophy (GA), or “dry” AMD, is characterized by regional loss of RPE and photoreceptors. Neovascular, or “wet” AMD, is characterized by choroidal neovascularization (CNV), which describes the growth of choroidal blood vessels into retina [1].

Pathologic changes that take place within the choriocapillaris and RPE following stress are believed to give rise to neovascular AMD. Angiogenesis, originating from the choriocapillaris, penetrates through Bruch's membrane and grows inward disrupting the overlying RPE and photoreceptors. These new vessels lack the structural integrity of established vasculature and exhibit incomplete basement membrane and limited pericytes. This gives rise to leakage of fluid and blood product into a region of the eye which is critical for fine vision and if left untreated, focal retinal detachment and loss of vision will ultimately ensue.

Whilst the damage caused by neovascular AMD arises from changes in the choriocapillaris, the key initiating factor is dysfunction of the RPE. The RPE has several specialized functions. That are central to the health of the retina including the secretion of vasoactive factors [29], phagocytosis of photoreceptor outer membranes [30], spatial buffering of ions [31], and epithelial transport to both the choriocapillaris [32] and the subretinal space [33]. A breakdown of the interplay between the RPE and the immunovascular system is thought to be the driving factor for CNV development [34].

The RPE vascular response is triggered by excess secretion of VEGF into the choroidal space [35]. This proangiogenic factor binds to receptors on endothelial cells [36] to initiate the process of CNV. Specifically, there are three types of VEGF receptors present on endothelial cells: VEGFR-1 (Flt-1), VEGFR-2 (KDR/Flk-1), and VEGFR-3 (Flt-4). Binding of VEGF to KDR/Flk-1 plays a key role in angiogenesis; Flt-1 functions as a decoy receptor, and Flt-4 is observed mostly in lymphatic vessels [37]. Several vasculogenic cytokines are also secreted by the RPE and contribute to the development of new vessels [38]. It is important to note that the RPE may not be the only source of proangiogenic factors.

One of the major pathways resulting in VEGF secretion from the RPE is in response to complement factors. The complement system, a component of the innate immune system, is a series of proteins that interact with one another to opsonize pathogens and mount an inflammatory response against infection. In recent years, numerous studies have found associations between sequence variants of complement pathway-associated genes and AMD [34, 39]. Complement has been found to be a constituent of drusen [40] and the presence of these proteins has the ability to induce excess VEGF production from the RPE [41] which works to disrupt epithelial tight junctions [42]. The deposition of complement in the retina is thought to occur secondary to oxidative stress, which is the oxidation of cellular macromolecules. Oxidative stress has been shown to reduce factors that inhibit complement deposition rendering cells susceptible to complement-mediated injury [42].

The immune system plays a role in the development and regulation of CNV and it appears to do so in a synergistic fashion in conjunction with the complement system. Complement factors C3a and C5a have been shown to be responsible for the recruitment of leukocytes to the choroid [41]. Macrophages are also upregulated and are a key feature of CNV lesions [43]. However, there is conflicting evidence as to whether their migration plays a protective role [44] or represents an exacerbation of disease [45]. Microglia may also play a role in the pathogenesis of CNV. In animal models, the accumulation of these immune cells in the subretinal space appears to amplify the effects of laser-induced CNV [46]. However, in human donor CNV specimens, a change in morphology of microglia is observed but with no increase in number [47]. There may also be a role for nonimmune cells in CNV development, as some one-third of all infiltrating cells in CNV are yet to be classified [48]. This underlines the need for further research into the role of the immune system in the pathogenesis of CNV.

### 2.2. Current Treatments for Neovascular AMD

Treatment for neovascular AMD has been revolutionised by the availability of intravitreal anti-VEGF agents. Such agents bind VEGF thereby preventing Flt-1 and KDR/Flk-1 signalling and inhibiting the neovascular response. In the treatment of classic CNV, Anti-VEGF agents have been shown to be superior to previous treatment modalities such as verteporfin photodynamic therapy [49, 50]. The most widely used drugs in the treatment of neovascular AMD are ranibizumab (a humanised antibody) and bevacizumab (an antibody fragment), and both bind and remove all bioavailable VEGF-A isoforms. Whilst ranibizumab has approval for ophthalmic use, bevacizumab is often administered “off-label” as a cost-effective alternative. These antibodies have a high affinity for VEGF-A and neutralise it, thus reducing receptor activation and suppressing endothelial cell proliferation and migration [51–53]. When compared, ranibizumab and bevacizumab show similar efficacy in inhibiting endothelial cell growth* in vitro* [54], although another study found ranibizumab was 11-fold more potent than bevacizumab at inhibiting endothelial cell proliferation [55].

Phase III studies (ANCHOR [56, 57] and MARINA [58]) have concluded that monthly administrations of ranibizumab 0.5 mg successfully inhibited the growth of CNV lesions. According to the Comparison of AMD Treatments (CATT) trial [4, 59], monthly injections of ranibizumab 0.5 mg prevented the loss of 15 letters in 94.4% of study participants over a 12-month period. The mean increase in BCVA was 8.5 early treatment diabetic retinopathy study (EDTRS) letters. For bevacizumab 1.25 mg administered via the same protocol, BCVA was stabilized in 94.0% of treated individuals with a mean improvement of 8.0 EDTRS letters.

More recently, aflibercept, a soluble decoy receptor protein with the capacity to neutralize all VEGF-A isoforms, was developed. Results of the VIEW 1 and 2 trials showed that the recommended aflibercept 2 mg treatment protocol (bimonthly injections after 3 monthly injections) was not inferior to ranibizumab 0.5 mg (monthly injections) after 12 months [60]. Further studies assessing the vision improvements and cost benefits of aflibercept over ranibizumab are required.

Anti-VEGF injections may be the standard mode of treatment for choroidal neovascularisation in AMD, but practitioners and patients must bear in mind that certain complications can arise from its administration. Results of the VEGF Inhibition Study in Ocular Neovascularization (VISION) clinical trial show that with intravitreal injection the incidence of endophthalmitis and retinal detachment was 0.16% and 0.08%, respectively [61]. For ranibizumab, the Anti-VEGF Antibody for the Treatment of Predominantly Classic Choroidal Neovascularization in Age-Related Macular Degeneration (ANCHOR) and Minimally Classic/Occult Trial of the Anti-VEGF Antibody Ranibizumab in the Treatment of Neovascular Age-Related Macular Degeneration (MARINA) study groups report presumed endophthalmitis in 1.0–1.4% of patients and serious uveitis in 0.7–1.3% [56, 58]. With all intravitreal injections there is also the possibility of damage to the crystalline lens during the procedure. The CATT study reported that the proportion of patients suffering from serious systemic adverse events was 24.1% in those treated with bevacizumab and 19.0% for ranibizumab [4]. Surprisingly, one study reports an 11% increase in all-cause mortality and a 57% increase in hemorrhagic stroke with intravitreal bevacizumab [8]. In contrast, a retrospective cohort study found no evidence for increased risks of mortality or stroke [7].

The Study of Ranibizumab in Patients with Subfoveal Choroidal Neovascularization Secondary to Age-Related Macular Degeneration (SUSTAIN) study assessed the efficacy of intravitreal ranibizumab for subfoveal choroidal neovascularisation secondary to AMD [62]. Patients were treated with 0.3 mg ranibizumab on a monthly basis for the first 3 months and then were treated on an “as needed” basis thereafter. Whilst 53% of patients respond well to the treatment and maintained their visual improvement over 12 months, 21% exhibited an initial increase in visual acuity for the first 3 months, followed by a steady decrease back to pretreatment levels. A decline in visual acuity with no response to therapy was observed in 26% of patients. At present, when a patient commences treatment with ranibizumab, there is no means to predict which group they may fall into and we do not understand what determines responder status.

These data show that whilst there is promise for an improvement in vision with intravitreal anti-VEGF agents, there are also shortcomings in terms of variable response to therapy as well as loss of efficacy in a subgroup of patients. In addition there are ocular as well as systemic secondary complications associated with the repeated intravitreal administration. Given the excessive costs to the healthcare system and burdens on the patient that have been eluded to earlier, there is a pressing need to look for new treatment modalities that might minimise complications, decrease frequency of administration, and decrease cost.

## 3. Gene Therapy and the Eye

In recent times, experimental work in gene therapy has gained momentum with many successes in treating both anterior and posterior eye disease. The basic premise of gene therapy involves implanting genetic material into host tissue in order to correct a dysfunctional gene or code for a therapeutic protein. Whilst gene therapy research typically targets monogenic degenerative diseases, there may be a role for gene therapy in multifactorial degenerative diseases such as diabetic retinopathy and age-related macular degeneration [63, 64].

There are numerous advantages of the eye as a target for gene therapy in comparison to other organs. Firstly, given that localised treatment can be performed instead of intravenous delivery, systemic absorption of gene vectors can be minimized. Once infected, the immune privileged state of the eye limits the provocation of unwanted systemic immune responses [65]. Furthermore, given that the eye consists of a comparatively small volume, minimal amounts of vector may be sufficient to achieve therapeutic levels of transgenes. Another advantage is the anatomy of the eye, which exhibits a high level of compartmentalization making specific cell populations easy to target. Finally, the transparent nature of the optical media permits ease of assessment by various techniques such as electroretinography, optical coherence tomography, and fundus fluorescein angiography.

A number of vectors are available for use in gene therapy; however, recombinant adenoassociated viruses (AAV) have shown great promise owing to their proven safety and exceptional expression kinetics. Belonging to the family Parvoviridae, these small, nonenveloped viruses comprise a linear single-stranded DNA genome. In the context of treating posterior eye diseases such as AMD, AAV vectors exhibit sustained transduction of the RPE, photoreceptors, and ganglion cells [66] with expression lasting several years [67]. Latent infection of AAV is set up due to integration of the virus into a specific locus on human chromosome 19 [68]. This implies that a single administration can offer longer-lasting treatment thereby reducing the need for multiple injections of anti-VEGF agents. What is more, AAV vectors do not induce inflammation or cytotoxicity [69] and studies in humans show negligible adverse effects [11].

Targeting specific cellular populations can be achieved with the advent of hybrid AAV vectors. These involve packaging the AAV plasmid of a particular serotype into the capsid of AAV from another serotype. For example, rAAV2/4 indicates a plasmid of serotype 2 has been encapsulated by that of serotype 4. Whereas rAAV2/4 produces gene expression limited to the RPE [67], rAAV2/7 and rAAV2/8 show promising transduction of photoreceptor cells [70]. Varying the plasmid/capsid serotype also has an effect on expression characteristics. In situations where rapid onset gene transfer is required, rAAV2/5 and 5/5 can produce expression in 3-4 days. If delayed onset is preferable, rAAV2/2 displays gradual levels of transduction efficiency until stable levels are reached in 2–4 months [71]. The repertoire of AAV vectors available can accommodate a wide range of tissue tropisms and expression profiles.

Lentiviral vectors are capable of long-term gene therapy in the eye and do so by integrating into the host genome. Such vectors are best at transducing nondividing cell populations such as the corneal endothelium, trabecular meshwork [72], and RPE [73]. The risk of viral replication via insertional mutagenesis is minimized through the use of highly deleted vectors [74] and self-inactivating vectors [75]. Examples of lentiviral vectors include human immunodeficiency virus-1 (HIV-1) and feline immunodeficiency virus (FIV).

Adenoviral vectors are nonintegrating and have the ability to transduce both dividing and nondividing cells. Gene expression is short-lived, however, due to elicitation of cytotoxic T lymphocyte-mediated immune responses [76]. A variety of nonviral vectors also exist such as DNA nanoparticles [77] and the *φ*C31 integrase system [78] and avoid the safety concerns associated with viral systems.

**Figure 1:**
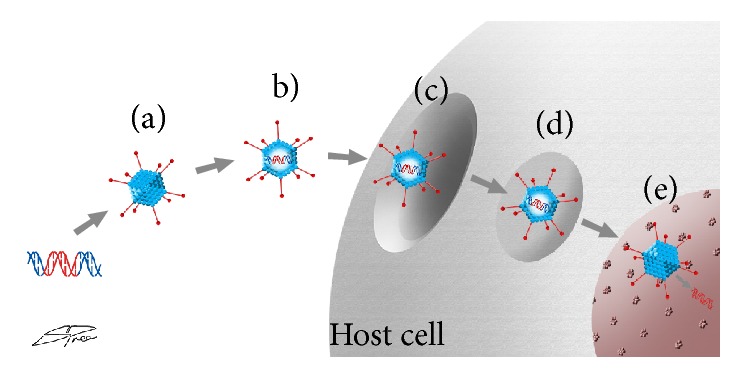
Schematic diagram of gene therapy with AAV. (a) Genetic material is biochemically engineered into the DNA of an AAV vector. (b) AAV is injected into the host. (c) Vesicle formation. (d) Internalization of AAV. (e) Breakdown of vesicle and delivery of genetic material to cell nucleus for protein production.

Figure 1 shows a schematic diagram of gene therapy. Genetic material is incorporated into the DNA of the AAV vector. It is then administered to the eye via a designated route which may be topical, subconjunctival, intracameral, intravitreal, or subretinal. The AAV plasmid/capsid combination is specifically selected to target the cellular population of interest. Once at the target cell, the vector attaches itself to membrane-bound receptors and becomes internalized via the formation of a vesicle. When it reaches the cell nucleus, the vesicle dissolves allowing the virus to deliver the genetic material for gene production.

Numerous clinical trials of gene therapy for retinal disease have been performed. The autosomal recessive disorder Leber's congenital amaurosis (LCA) is in Phase III trials with promising results. Improvements to dark-adapted function and pupillary light reflexes were noted. Most importantly, no significant changes were observed in visual acuity, visual field, or electroretinogram response after exposure to the rAAV2 vector [11].

## 4. Targeting VEGF via Gene Therapy

Whilst the underlying mechanisms leading to the development of CNV are not fully understood, it is clear that inhibition of VEGF and its receptor is quite effective at arresting choroidal neovascularisation. The next generation of treatment for neovascular AMD must demonstrate wider and longer-term efficacy and reduce the need for frequent administrations, hence reducing costs. It is also imperative that adverse reactions to the treatment are minimized. Whilst still in its experimental and early clinical trial stages, gene therapy appears to possess all of the characteristics necessary to improve upon the current intravitreal anti-VEGF treatment modality.

Animal studies have shown that VEGF over expression can be arrested using gene therapy. Whilst intravitreal gene transfer of antiangiogenic agents has proved to be successful in suppressing experimental CNV [79], the test subject remains at risk of adverse reactions that may arise from invasive intravitreal injections. To overcome this issue, topical administration of angiogenic inhibitors has been trialled and have shown some success in reducing CNV lesions induced by laser rupture of Bruch's membrane [80]. There was, however, the need for a high rate of administration of three times a day, which raises concerns of compliance and systemic absorption via the nasal mucosa. Subconjunctival gene transfer might provide a more localised but less invasive delivery route compared with intravitreal injections and at the same time would negate the need for frequent eye drops and avoid mucosal absorption.

Although anti-VEGF gene therapy provides a way to avoid the limitations of conventional therapy by intravitreal anti-VEGF agents, the issue of systemic safety from long-term neutralization of VEGF remains a concern. This is of particular importance given reports of increased risk of hemorrhagic stroke and RPE atrophy. Therefore, in addition to the development of new delivery routes for gene-based delivery of anti-VEGF agents, there is an intensive search for alternative antiangiogenic agents. Advances in this area are reviewed in the following section.

## 5. Potential Endogenous Inhibitors of Angiogenesis for Gene Therapy

Angiogenesis is dynamically regulated by the interplay of proangiogenic and antiangiogenic factors. Physiologically, the balance is skewed towards angiogenic inhibitors so that unwanted angiogenesis does not occur [81]. However, this state of homeostasis is disturbed in pathological conditions like neovascular AMD where angiogenic factors counterbalance endogenous inhibitors leading to the aberrant growth of leaky blood vessels. Expression of endogenous inhibitors including PEDF [82] and endostatin [83] in RPE and Bruch's membrane has been found to be reduced in choroid samples from donors affected by AMD. Further immunohistochemical characterisation reveals a decrease in other endogenous inhibitors such as thrombospondin-1 in the RPE, Bruch's membrane, and choriocapillaris [84] where AMD pathology occurs. This suggests that an accumulation of endogenous inhibitors in RPE—Bruch's membrane—choriocapillaris complex could act as a protective barrier for stopping the intrusion of new blood vessels [84]. Apart from suppressing angiogenesis, these inhibitors possess other useful biological functions that make them appealing for gene therapy (Table 1).

### 5.1. Pigment Epithelium-Derived Factor (PEDF)

PEDF belongs to the serine protease inhibitor family and was first isolated from fetal human RPE cells [85]. It is extensively expressed throughout various layers of the human eye including the ciliary epithelium, inner and outer retina, and cornea [85]. Its expression is found to be altered in eyes affected by AMD, specifically in regions where AMD pathology is actively occurring [82]. PEDF is advantageous as a potential target over other endogenous inhibitors due to its neurotrophic and neuroprotective properties. In addition to its antiangiogenic effect on endothelial cells, PEDF has been shown to promote the survival of neuronal cells, preserve their integrity, and protect them from apoptosis [85]. Gene transfer using adenovirus based vectors in mice can successfully produce ocular levels of PEDF protein well above the therapeutic threshold. In one study this led to a regression in oxygen-induced retinal neovascularisation [18], demonstrating the efficiency and efficacy of adenovirus mediated gene transfer. Safety issues were recently addressed by a Phase 1 clinical study, which explored the safety and efficiency of an intravitreal injection of two different titres of an adenovirus vector expressing PEDF in twenty-eight patients with advanced neovascular AMD over 12-month period [21]. A quarter of patients shows mild transient ocular inflammation and six subjects exhibited manageable elevated intraocular pressure [21]. Therefore, gene transfer of PEDF in patients is well-tolerated. Although therapeutic efficacy was not the objective of Phase 1 study, 50% of patients treated with the higher titre of PEDF expressing vector showed a reduction in lesion size at 6 and 12 months following treatment. This is evidence of an extended antiangiogenic effect following a single injection [21]. There have been no further clinical studies on PEDF gene transfer but a recent animal study demonstrated an anti-inflammatory action of recombinant PEDF protein in mice with spontaneous retinal degeneration [86]. Therefore the versatile biological functions of PEDF make it an attractive target for gene transfer therapy.

### 5.2. Angiostatin

Angiostatin is a cleaved product of plasminogen containing the kringle domains 1–4. It has well-characterised antiangiogenic effects and its therapeutic potential arises from its effectiveness in studies of tumour treatment [87]. Angiostatin promotes apoptosis of proliferating vascular endothelial cells [88] and inhibits proliferation and migration of endothelial cells [89]. The importance of angiostatin in suppressing the growth of retinal neovessels has been documented in a study showing that the local release of angiostatin is an important factor mediating the beneficial action of laser photocoagulation in patients with proliferative diabetic retinopathy [90]. In a murine model of proliferative diabetic retinopathy lentivirus-mediated expression of angiostatin was shown to be a potent inhibitor of neovascularisation [91]. Moreover, systemic administration of recombinant angiostatin in neonatal mice inhibits ischemia-induced growth of retinal vessels with little effect on the normal process of retinal vessel development [92]. This illustrates its selectivity for suppressing pathological and not normal angiogenesis. One study used an adenoviral vector to overexpress kringle domains 1–3 of angiostatin in the neonatal mouse retina. Results showed inhibition of ischemia-induced neovascularisation, as reflected by a marked reduction in the number of endothelial cells in the retinal layer where neovascular tufts originate [18]. Inhibitory effects of the transgene correlated well with ocular protein expression since its level was found to be well above the therapeutic threshold. Importantly, administration of the adenoviral vector did not result in cytotoxicity [18], highlighting the clinical potential of gene delivery with angiostatin. The highly stable lentivirus-based vector has also been used to deliver angiostatin to rat eyes with an observable decrease in the area of experimental choroidal neovascularisation [16]. Angiostatin has also been shown to suppress the recruitment and adhesion of inflammatory cells to the endothelium, in addition to limiting their transmigration [93]. A 6-month safety study of lentiviral gene delivery of angiostatin in rhesus macaques and rabbits found no change in retinal functional, as evaluated by electroretinography, and no histological structural changes [94]. In summary, angiostatin transgene has significant appeal as a viable therapeutic approach.

### 5.3. Endostatin

Like angiostatin, endostatin is a potential therapeutic target for treatment of tumour growth owing to its antiangiogenic properties [95]. One such mechanism involves its interaction with VEGF. Endostatin has been shown to prevent the binding of VEGF to its receptor KDR/Flk-1 in endothelial cells [96]. Endostatin also inhibits the spontaneous release of VEGF from human endothelial cell culture [97]. Moreover, endostatin has been shown to suppress VEGF-mediated responses* in vivo* [98]. Lentiviral-mediated overexpression of endostatin in the mouse retina reduced the degree of neovascularisation and vascular leakage, which were both stimulated by locally expressed VEGF transgene [98]. Adenoviral-mediated expression of endostatin was shown to be successful in inhibiting neovascular responses in a mouse model of retinopathy of prematurity [99]. Proapoptotic activity of endostatin also contributes to its antiangiogenic properties. Lentiviral delivery of endostatin induced a decrease in the extent of choroidal neovascularisation, vascular hyperpermeability, and apoptotic cell loss in the neurosensory retina of laser damaged rat eyes [16]. Immunohistochemical characterisation confirms that the proapoptotic activity of the endostatin transgene in neurosensory retina is limited to the laser-damaged eye [16]. This underlines the selectivity of endostatin against pathological growth of vessels. Safety studies have shown no change in retinal structure and function following lentiviral gene therapy with endostatin [94].

Endostatin also has a structural support role, which makes it valuable in gene therapy. It is a proteolytic fragment of collagen XVIII and forms a crucial component of Bruch's membrane [100]. Deletion of endostatin in mice causes a phenotypic change including morphological abnormality of the RPE with an accumulation of sub-RPE deposit formation in the Bruch's membrane that contributes to age-dependent vision loss [100]. Such findings correlate with a reduced expression of endostatin found in Bruch's membrane of human AMD sufferers [83], indicating a requirement of endostatin for a functional Bruch's membrane. Targeted gene therapy with endostatin is therefore a promising therapeutic strategy.

### 5.4. Tissue Inhibitor of Metalloproteinases-3 (TIMP-3)

TIMP-3 is an extracellular matrix component of Bruch's membrane [101] synthesized by the RPE, choroid and retina [17]. It is the only member of the peptidases that is distributed in the extracellular matrix of the membrane, where it regulates the proteolytic activity of matrix metalloproteinases. The unique location of TIMP-3 suggests a physiological role at the interface of the RPE, Bruch's membrane, and choroid [102]. Indeed mice with a deficiency of TIMP-3 exhibit abnormal development of blood vessels characterised by dilated capillaries at the choroid and augmented activity of matrix metalloproteinases [102]. The abnormal choroidal vascular network in TIMP-3 knockout animals may also be related to the imbalance of angiogenic homeostasis [102] given that TIMP-3 has been shown to possess antiangiogenic activity [103]. Overexpression of TIMP-3 in the eye using gene delivery produces a reduction in laser-induced choroidal neovascularisation [17] and ischemia-induced retinal neovascularisation [99] in rats and mice. Whereas endostatin inhibits the binding of VEGF to KDR/Flk-1, TIMP-3 selectively binds to KDR/Flk-1 but not to Flt-1 [103].

### 5.5. Vasostatin

Vasostatin is a naturally occurring peptide found in humans and is derived from the NH_2_ terminal domain of a calcium-binding protein calreticulin [22]. Recombinant vasostatin has been shown to inhibit the proliferation of human endothelial cells, stimulated by basic fibroblast growth factor (bFGF) [22]. Topical application of recombinant protein to rats subjected to laser photocoagulation also causes a reduction in the area of choroidal lesions [80], underlining its therapeutic potential for suppressing neovascularisation. It has been postulated that the antiangiogenic effect may be due to interference with the signaling of the controversial regulator of angiogenesis angiopoietin 2 [104]. Vasostatin is found to reduce the expression of angiopoietin 2 in inflamed skin, which mediates inflammatory responses including formation of blood vessels, infiltration of inflammatory cells, and adherence of leukocytes to endothelium [104]. Angiopoietin 2 destablilizes blood vessels and has been shown to disrupt early proliferating vessels thereby promoting vessel maturation [105]. Angiopoietin 2 can also induce angiogenesis via the binding of integrins in activated endothelial cells that have a diminished population of Tie2 receptors [106]. The inhibitory mechanism of angiopoietin 2 may explain the selective antiangiogenic effects of vasostatin on endothelial cells of proliferating vessels.

### 5.6. Plasminogen Kringle 5 (K5)

K5 is derived from plasminogen and its antiangiogenic activity appears to be specific for endothelial cells as it inhibits proliferation and migration and promotes apoptosis [107, 108]. Recombinant K5 only suppresses the proliferation of endothelial cells but not vascular smooth muscle cells or fibroblasts under the stimulation of VEGF [108]. When recombinant K5 is given locally via intravitreal injection, either before or during the development of oxygen-induced retinal neovascularisation in rats, the degree of neovascularisation is suppressed [23]. Importantly it reduces the number of vascular endothelial cells in proliferating vessels but not preexisting vessels of rats with oxygen-induced retinopathy [23], supporting its selective action against pathological angiogenesis. K5 may restore angiogenic homeostasis to exert an antiangiogenic effect. Indeed an intravitreal injection of K5 decreases the retinal expression of VEGF while it elevates PEDF in rats with oxygen-induced retinopathy [107]. An interference with the autophagy phase of apoptotic endothelial cells may also contribute to its antiangiogenic activity [109]. Other useful biological actions of K5 include antihyperpermeability and anti-inflammation. Recombinant K5 given through either systemic or ocular administration reduces the extent of retinal vascular leakage in both rat models of oxygen-induced retinopathy and streptozotocin-induced diabetes [110]. The antihyperpermeability effect of recombinant K5 could be related to a reduction in retinal expression of VEGF, which has been shown to cause hyperpermeability in both models [110]. Topical administration of recombinant K5 has also been found to suppress alkali-induced neovascularisation, infiltration of inflammatory cells, and VEGF expression in the rabbit cornea [108], indicating its effectiveness in hampering an inflammation-driven angiogenic response. In addition, nanoparticle-mediated transfer of K5 in the rat retina has been shown to produce an inhibitory effect on experimental CNV [111].

### 5.7. Thrombospondin-1

Thrombospondin-1 belongs to the glycoprotein family and regulates the structure of extracellular matrix and cellular phenotype associated with tissue remodelling during angiogenesis [112]. The expression of thrombospondin-1 in RPE, Bruch's membrane, and the choriocapillaris in human AMD choroids is found to be less than that of controls [84], suggesting a protective role of thrombospondin-1 in AMD. One of the antiangiogenic effects of thrombospondin-1 appears to be mediated by an induction of apoptotic endothelial cells. Indeed knocking down the expression of thrombospondin-1 in mice resulted in a two-fold decrease in the number of apoptotic nuclei in developing retinal vessels [113]. An increased count of retinal endothelial cells as an index of retinal vascular density is also demonstrated in mice lacking thrombospondin-1 [113]. Moreover, Sorenson et al. [114] recently induced a deletion of thrombospondin-1 in Akita mice that develop spontaneous diabetes and showed an acceleration of diabetes-induced retinopathy in the absence of thrombospondin-1. Collectively, thrombospondin-1 is required for a quiescent and differentiated phenotype of endothelial cells [113]. It is unclear whether an overexpression of thrombospondin-1 in eyes exerts a protective effect against neovascular AMD; however, its anti-inflammatory action [115] will be valuable for suppressing aberrant vessel growth. Therefore, gene transfer studies in animals are warranted to examine a role of thrombospondin-1 in neovascular AMD.

## 6. Conclusion and Future Perspective

Gene therapy shows great promise in the treatment of eye disease and the prevention of blindness. It is much easier and less costly to manufacture gene therapy vectors than to produce huge amounts of purified protein molecules. The recent data from animal studies and Phase I clinical trials has indicated that gene therapy of anti-VEGF agent such as sFlt-1, a soluble form of the Flt-1 receptor, provided major benefits in patients with neovascular AMD and other types of ocular neovascularization. These data suggest that long term blockade of VEGF in the retina and choroid by gene transfer is likely to inhibit neovascularization, but it is not yet known if sustained, efficient blockade of VEGF family members will have any adverse effects on normal choroidal vessels and retinal neurons. Moreover, similar to protein-based anti-VEGF treatments, the loss of efficacy of anti-VEGF gene therapy is a clinically significant problem in the battle against neovascular AMD. Thus, an alternative gene-based approach with expression of one or more of the aforementioned endogenous angiogenic inhibitors has excellent potential. Most of the endogenous angiogenic inhibitors have a small molecular size, specifically target endothelial cells, and are effective in preventing the development of neovascularization with no effect on established vessels. Gene transfer of PEDF produced beneficial effects in animal models and a Phase I study has shown an excellent safety profile for intraocular injection of Ad-PEDF. Although therapeutic efficacy is not an objective of Phase 1 studies, patients who received the treatment showed a reduction in lesion size. Moreover, a Phase I single dose trial with a lentiviral vector-mediated expression of two angiogenic inhibitors, endostatin and angiostatin (retinostat), has recently commenced in neovascular AMD patients. Other promising candidates with antiangiogenic properties include vasostatin, TIMP3, K5, and thrombospondin-1. Numerous studies have demonstrated their therapeutic effects, however; further gene transfer studies in animals are needed to build the basis for clinical translation.

## Figures and Tables

**Table 1:** Biological actions of endogenous inhibitors of angiogenesis.

Endogenous inhibitor	Functions
**PEDF**, serine protease	Increases survival of neuronal cell, preserves the integrity of neuronal cells; protects neuronal cells from apoptosis, decreases proliferating endothelial cells, decreases expression of inflammatory molecules like TNF*α* and iNOS.

**Angiostatin**, cleaved product of plasminogen containing the kringle domains 1–4	Increases apoptosis of proliferating vascular endothelial cells, decreases proliferation and migration of endothelial cells, decreases recruitment and adhesion of inflammatory cells to the endothelium, and decreases transmigration of inflammatory cells.

**Endostatin**, fragment of collagen XVIII	Increases apoptosis and decreases migration of cells involved in active neovascularisation, blocks the binding of VEGF to KDR/Flk-1, and decreases spontaneous release of VEGF from endothelial cell culture, structurally supports role of the Bruch's membrane.

**TIMP3**, inhibitor of matrix metalloproteinase	Increases apoptosis and decreases migration of cells involved in active neovascularisation, blocks the binding of VEGF to KDR/Flk-1, structurally supports role of the Bruch's membrane.

**Vasostatin**, a derivative from the NH_2_ terminal domain of a calcium binding protein calreticulin	Decreases proliferation of endothelial cells, decreases adhesion of leukocytes to endothelium, decreases expression of vascular destabilising factor angiopoietin 2.

**Plasminogen kringle 5**, cleaved product of plasminogen containing the kringle domain 5	Increases proliferation and decreases migration of endothelial cells, increases apoptosis of endothelial cells, increases infiltration of inflammatory cells.

**Thrombospondin-1**, glycoprotein	Decreases apoptosis of endothelial cells, decreases expression of inflammatory molecules.
